# Effects of diets high in animal or plant protein on oxidative stress in individuals with type 2 diabetes: A randomized clinical trial

**DOI:** 10.1016/j.redox.2019.101397

**Published:** 2019-11-28

**Authors:** Olga Pivovarova-Ramich, Mariya Markova, Daniela Weber, Stephanie Sucher, Silke Hornemann, Natalia Rudovich, Jens Raila, Daniele Sunaga-Franze, Sascha Sauer, Sascha Rohn, Andreas F.H. Pfeiffer, Tilman Grune

**Affiliations:** a Dept. of Clinical Nutrition, German Institute of Human Nutrition Potsdam-Rehbruecke, Nuthetal, Germany; b Reseach Group Molecular Nutritional Medicine, Dept. of Molecular Toxicology, German Institute of Human Nutrition Potsdam-Rehbruecke, Nuthetal, Germany; c German Center for Diabetes Research (DZD), München-Neuherberg, Germany; d NutriAct-Competence Cluster Nutrition Research Berlin-Potsdam, Nuthetal, Germany; e Department of Molecular Toxicology, German Institute of Human Nutrition Potsdam-Rehbruecke (DIfE), Nuthetal, Germany; f Dept. of Endocrinology, Diabetes and Nutrition, Campus Benjamin Franklin, Charité University of Medicine, Berlin, Germany; g Division of Endocrinology and Diabetes, Department of Internal Medicine, Spital Bülach, Bülach, Switzerland; h Institute of Nutritional Science, University of Potsdam, Nuthetal, Germany; i Max Delbrück Center for Molecular Medicine (MDC) in the Helmholtz Society, Berlin, Germany and Berlin Institute of Health, Berlin, Germany; j Institute for Food and Environmental Research e.V, Bad Belzig, Germany; k Institute of Food Chemistry, Hamburg School of Food Science, University of Hamburg, Hamburg, Germany; l German Center for Cardiovascular Research (DZHK), Berlin, Germany

**Keywords:** Animal protein, Plant protein, Type 2 diabetes, Oxidative stress, Antioxidant status

## Abstract

High-protein diet is a promising strategy for diabetes treatment supporting body weight control, improving glycaemic status, cardiovascular risk factors and reducing liver fat. Here, we investigated effects of diets high in animal (AP) or plant (PP) protein on oxidative stress and antioxidant status in individuals with type 2 diabetes (T2DM). 37 obese individuals (age 64.3 ± 1.0 years) with T2DM were randomized to an isocaloric diet (30 energy(E)% protein, 30 E% fat and 40 E% carbohydrates) rich in AP or PP for 6 weeks. Markers of oxidative and nitrosative stress and antioxidant status in plasma and nitrate/nitrite levels in urine were assessed. Gene expression in subcutaneous adipose tissue (SAT) was analysed by RNA-Seq and real-time PCR. Both AP and PP diets similarly reduced plasma levels of malondialdehyde (P_AP_ = 0.003, P_PP_ = 1.6 × 10^−4^) and protein carbonyls (P_AP_ = 1.2 × 10^−4^, P_PP_ = 3.0 × 10^−5^) over 6 weeks. Nitrotyrosine (NT) increased upon both AP and PP diets (P_AP_ = 0.005, P_PP_ = 0.004). SAT expression of genes involved in nitric oxide (NO) and oxidative stress metabolism and urine NO metabolite (nitrate/nitrite) levels were not changed upon both diets. Plasma levels of carotenoids increased upon PP diet, whereas retinol, alpha- and gamma-tocopherol slightly decreased upon both diets. AP and PP diets similarly improve oxidative stress but increase nitrosative stress markers in individuals with T2DM. Mechanisms of the NT regulation upon high-protein diets need further investigation.

## Highlights

• Effects of 6-week isocaloric diets high in animal (AP) or plant (PP) protein in subjects with type 2 diabetes were studied.
• AP and PP diets reduced plasma levels of malondialdehyde and protein carbonyls and increased nitrotyrosine.
• Expression of genes of nitric oxide (NO) and oxidative stress metabolism and urine NO metabolite levels were not changed.
• Plasma retinol and tocopherol decreased upon both diets, and carotenoids increased upon PP diet.

## Graphical abstract

**Figure:**
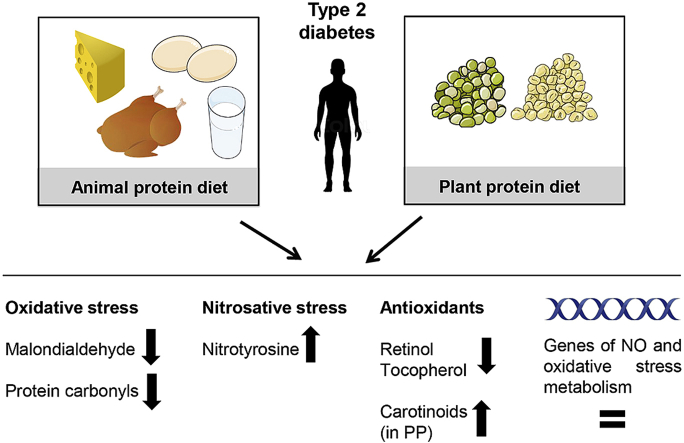

## Abbreviations

AP: Animal protein
CID: Clinical investigation day
E%: Per cent of energy
FFA: Free fatty acids
HOMA-IR: Homeostasis model assessment of insulin resistance
MDA: Malondialdehyde
MUFA: Monounsaturated fatty acids
NEFA: Non-esterified fatty acids
NO: Nitric oxide
NT: Nitrotyrosine
PP: Plant protein
PUFA: Polyunsaturated fatty acids
RNS: Reactive nitrogen species
ROS: Reactive oxygen species
SFA: Saturated fatty acids
TBA: thiobarbituric acid
T2DM: Type 2 diabetes mellitus

## 1 Introduction

Nutritional strategies have been shown to be highly effective to improve metabolic control in individuals with type 2 diabetes (T2DM), but there are controversies regarding the question which dietary composition is more efficacious. We and others already showed that high-protein diets effectively support weight loss and weight maintenance [1] due to increased satiety and postprandial thermogenesis. Although epidemiological studies demonstrate association of high intake of red meat and animal protein in general with increased risk of T2DM [2], clinical studies with T2DM patients observed strong improvement of glycaemic control and reduction of HbA1c after both animal and plant protein diets [[3], [4], [5]]. High-protein diets also potently reduce liver fat, improve blood lipid profile, and decrease blood pressure [3,6,7], suggesting that high-protein diet is a promising strategy for diabetes prevention and treatment. However, there are limited data on the effects of high-protein diets on the oxidative and nitrosative stress in humans [8,9].

T2DM is characterized by the elevated oxidative and nitrosative stress which result from overproduction and/or decreased elimination of reactive oxygen species (ROS) and reactive nitrogen species (RNS) and are linked to the development of insulin resistance, cardiovascular complications, and chronic low-grade inflammation [10,11]. Various causative factors are suggested to contribute to the increased generation of ROS and RNS in T2DM including hyperglycemia, FFA elevation, and overnutrition caused by excess high-fat and/or carbohydrate diets [10,12]. Enhanced ROS production stimulates lipid peroxidation and protein carboxylation leading to the dysfunction of multiple cellular components and metabolic pathways [12]. Hyperglycemia also induces increased nitric oxide (NO) production [13], which interacts with ROS to generate peroxynitrite (ONOO^−^) inducing nitrosative stress and disrupting cellular signaling and metabolism [11]. Overall, enhanced oxidative damage is one of major mechanisms of glucose toxicity in T2DM and its related micro- and macrovascular complications.

As mentioned above, nutrient composition and calorie intake could have a large effect on the redox status, strongly affecting levels of pro- and antioxidant factors in plasma [14]. In particular, Kitabchi et al. found that a hypocaloric high-protein diet more effectively reduce ROS levels and lipid peroxidation in comparison with a high-carbohydrate diet accompanied by stronger improvement of insulin sensitivity, cardiovascular risk factors and inflammatory cytokines [8]. Whether dietary protein of animal or plant origin can differently modulate oxidative stress and antioxidant status still remains to be elucidated. We therefore compared effects of isocaloric diets high in animal or plant protein in individuals with T2DM in a 6-week randomized clinical trial.

## 2 Materials and methods

### 2.1 Study design and dietary intervention

Individuals with diagnosed T2DM and HbA_1c_ ≥ 6% were included in this randomized open-label, parallel-arm clinical trial. Study design was approved by the Ethics Committee of the University of Potsdam, conducted in accordance with the Declaration of Helsinki, and registered at www.ClinicalTrials.gov (NCT02402985). All participants provided written informed consent before starting the study. Details of the study design, inclusion and exclusion criteria as well as dietary intervention were published previously [3,6].

In brief, 44 individuals with type 2 diabetes were assigned to a high protein diet from either plant (PP) or animal (AP) origin; randomizing parameters were age, sex, BMI, HbA_1c_ level, and oral medication. Macronutrient intake of individuals prior to enrollment was 17 E% protein, 42 E% carbohydrates (CHO), 41 E% fat. 37 subjects (nAP = 18, nPP = 19) in age of 64.3 ± 1.0 years completed the study (Fig. S1). Both diets were isocaloric and had the same macronutrient composition (30 E% protein, 40 E% CHO, 30 E% fat consistent of 10 E% saturated, 10 E% monounsaturated, 10 E% polyunsaturated fatty acids) (Table 1). The animal-protein diet (AP) was rich in meat and dairy foods, the plant-protein diet (PP) consisted mainly of pea protein. In order to achieve good compliance, maximal amount of AP in the PP diet was around 28%; PP content in the AP diet was limited to 20% of protein intake. Study participants received individual isocaloric food plans created based on the individual basal metabolic rate, total energy expenditure and individual preferences as described previously [3,6]. Participants were advised to weigh and document all foods they had eaten including aberrations from the food plans. Analysis of food plans was performed with PRODI® 6.2 (Nutri-Science GmbH, Hausach, Germany) which included the Bundeslebensmittelschlüssel (BLS), version 3.01. Glycemic index and fiber content was similar in both groups. Participants were asked to maintain their physical activity patterns for the duration of the study.

**Table 1:** Dietary intake of macronutrients and vitamins.

Variables		AP		PP	
		Week 0	Week 6	Week 0	Week 6
Energy (kJ/day)		9525.2 ± 421.2	10458.3 ± 378.5*	9136.0 ± 482.4	9784.2 ± 466.0*
Protein	(E%)	17.6 ± 0.7	29.5 ± 0.2**	16.4 ± 0.6^##^	29.9 ± 0.2**
	(g)	96.1 ± 6.3	178.3 ± 6.0**	81.8 ± 4.6	169.1 ± 8.4**
Plant protein (%)			19.8 ± 0.3		72.3 ± 0.9^##^
Animal protein (%)			80.2 ± 0.3		27.7 ± 0.9^##^
Carbohydrates	(E%)	41.3 ± 1.3	40.5 ± 0.2**	43.3 ± 1.4^##^	39.3 ± 0.3**^##^
	(g)	222.9 ± 9.8	244.6 ± 8.2**	214.3 ± 10.0	222.3 ± 11.2
Fat	(E%)	41.1 ± 1.2	30.1 ± 0.2**	40.3 ± 1.2^##^	30.9 ± 0.4**
	(g)	102.5 ± 7.0	81.9 ± 2.4**	91.7 ± 5.2	78.3 ± 3.3*
SFA (g)		42.8 ± 3.2	27.63 ± 0.92**	37.6 ± 2.3	24.08 ± 1.05**^#^
MUFA (g)		15.6 ± 1.4	25.18 ± 0.85**	14.7 ± 1.2	23.88 ± 1.10**
PUFA (g)		36.9 ± 2.7	23.66 ± 1.14**	33.0 ± 2.3	23.38 ± 1.06**
			54.5 ± 0.4		55.7 ± 1.3
Dietary fibre (g)		25.5 ± 2.0	35.36 ± 1.42**	26.3 ± 1.6	33.27 ± 1.78**
Retinol (μg)		1123.4 ± 212.1	378.4 ± 18.4**	1162.9 ± 571.2	372.7 ± 35.1**
Beta-carotene (μg)		5818.2 ± 1668.6	9460 ± 641**	5198.9 ± 793.1	14190 ± 1063**^##^
Vitamin E (mg)		11.9 ± 1.0	21.3 ± 0.8**	14.0 ± 1.2	21.3 ± 1.2**
Vitamin C (mg)		142.4 ± 22.3	276.8 ± 13.6**	194.9 ± 25.8	258.3 ± 20.0**

Values are means ± SEM. n_AP_ = 18, n_PP_ = 19. *p < 0.05, **p < 0.01 week 6 vs. week 0; #p < 0.05, ##p < 0.01 AP vs. PP at week 0 or at week 6.

At the beginning and end of interventions, hyperinsulinemic euglycemic clamps were performed after overnight fast as described [3,6]. In the hyperinsulinaemic–euglycaemic clamp, whole-body insulin sensitivity (M-value, insulin-mediated glucose uptake per kg body weight) was determined by at a constant insulin infusion rate of 40 mU/kg/min.

### 2.2 Body composition

Body composition was determined by air displacement plethysmography (BOD POD; Cosmed, Rome, Italy).

### 2.3 Biomarker analysis

Blood was collected at week 0, 2, 4, and 6 of the intervention after overnight fast. Blood samples were immediately chilled and centrifuged, and the supernatant was stored at −80 °C until analysed. Routine laboratory markers were measured by using standard methods (ABX Pentra 400, ABX Diagnostics, Montpellier, France). ELISAs were performed to determine serum levels of insulin (Mercodia, Uppsala, Sweden), adiponectin (R&D Systems, Minneapolis, MN), and IL-6 (Merck Sharp & Dohme, Kenilworth, NJ). Tumor necrosis factor alpha (TNFα) was measured using Luminex magnetic bead technology (R&D Systems, Minneapolis, MN). Index of whole-body insulin resistance (HOMA-IR) was calculated as: fasting insulin [μU/mL] x fasting glucose in [mM]/22.5.

Malondialdehyde (MDA) was measured in plasma samples before and 180 min after each MTT as a marker of lipid peroxidation after derivatization with thiobarbituric acid (TBA) and separation by reverse-phase HPLC coupled with fluorescence detection (free MDA as standard) as described [15,16]. The analyses of nitrotyrosine, protein carbonyls [16] and micronutrients [17] in plasma has been described in detail elsewhere.

Plasma amino acid levels were determined by liquid chromatography tandem mass spectrometry analysis as described recently [6]. Nitrate and nitrite levels in 24-h urine samples were determined using Nitrate/Nitrite Colorimetric Assay Kit (Cayman Chemical, Ann Arbor, MI).

### 2.4 Gene expression analysis of adipose tissue

Subcutaneous adipose tissue (SAT) samples were obtained from 27 subjects by fine-needle biopsy before and after 6 weeks of dietary intervention. Samples were flash-frozen in liquid nitrogen and stored at −80 °C until analysis.

For the gene expression analysis by qPCR, total RNA was purified from SAT samples using the miRNeasy Lipid Tissue Mini Kit (Qiagen, Germany). RNA concentration was measured using an ND-1000 spectrophotometer (Nanodrop, PeqLab). Single-stranded cDNA was synthesized with miScript II RT Kit (Qiagen, Germany). QPCR was performed by ViiA 7 sequence detection system using Power SYBR Green PCR Master Mix (Applied Biosystems, USA) and specific primers. Gene expression was assessed by the standard curve method and normalized to the reference gene beta-glucuronidase (GUSB). Primer sequences are shown in Table S1.

For mRNA-sequencing, mRNA was performed using the stranded mRNA library preparation kit from New England Biolabs. Paired-end sequencing of 75 nt was performed using an Illumina HiSeq 4000 sequencer. The reads were mapped using bowtie v.2.3.2 [18] and GENCODE human reference genome (GRCh38. p10). Once reads were mapped, they were counted using RSEM v. 1.3 software [19]. The differential expression of mRNA isoforms was evaluated with DESeq2 using the paired experimental design (p-value ≤ 0.05) [20]. The transcript annotation of the mRNA isoforms was retrieved from the BioMart database [21] and functional annotation was done using ConsensusPathDB [22]. The transcriptome data can be found under EBI Annotare v.2.0 (Project-ID: E-MTAB-8549).

### 2.5 Statistical analysis

Data are presented as the mean ± SEM. Statistical significance was defined as *p* < 0.05. Non-normaly distributed variables were transformed with the natural logarithm and re-assessed for normality. Comparisons between two groups were tested by Student's *t*-test (paired and unpaired) or non-parametric tests (Wilcoxon and Mann-Whitney-U-Test). Repeated measures ANOVA was used for comparisons within and between dietary groups. Depending on data distribution, Pearson's coefficient or Spearman's rank correlation coefficient was used for correlation analysis. All statistical analyses were performed with SPSS 20.0 (Chicago, USA).

## 3 Results

### 3.1 Clinical characteristics of participants

A total of 37 participants completed the intervention (AP n = 18, PP n = 19). Subjects were 65.0 ± 1.4 (AP) years old and 63.7 ± 1.5 (PP) years old, moderately obese with BMI of 31.0 ± 0.8 kg/m^2^ (AP) and 29.4 ± 1.0 kg/m^2^ (PP) and with well controlled HbA1c (at ~7.0% in both groups). Both AP and PP diet induced slight decrease of BMI (AP: −2.6%; PP: −1.7%) and fat mass (AP: −1.9%; PP: −1.4%) without difference between the groups (Table 2). After 6 weeks of intervention, high-protein diet markedly improved glycemic control, i.e. decreased HbA1c in both groups and fasting glucose and whole-body insulin sensitivity in the AP group (Table 2). Blood lipids, i.e. total cholesterol, HDL and LDL cholesterol, were reduced in both groups, whereas NEFA decreased significantly only in the PP group. Inflammatory marker C-reactive protein (CRP) decreased upon AP diet, whereas TNFα decreased in the PP group (Table 2).

**Table 2:** Characteristics of the study subjects.

Parameter	AP			PP			AP versus PP
	week 0	week 6	*p*_*AP*_	week 0	week 6	*p*_*PP*_	*p*_*APvsPP*_
Age (years)	65.0 ± 1.4			63.7 ± 1.5			
Body mass index (kg/m^2^)	31.0 ± 0.8	30.2 ± 0.7	1.4*10^**−4**^	29.4 ± 1.0	28.9 ± 1.0	**0.005**	0.088
Fat mass [%]	35.26 ± 2.19	33.36 ± 1.94	**0.023**	34.95 ± 2.30	33.55 ± 2.20	0.107	0.473
Fasting insulin (mU/l)	10.07 ± 1.69	8.31 ± 1.27	0.701	8.74 ± 1.32	9.12 ± 1.72	1.000	0.869
Fasting glucose (mmol/l)	9.64 ± 0.43	8.61 ± 0.36	**0.043**	9.48 ± 0.35	9.35 ± 0.50	0.242	0.138
HOMA-IR	4.45 ± 0.87	3.15 ± 0.49	0.183	3.82 ± 0.53	3.67 ± 0.61	0.494	0.767
HbA_1c_ (%)	6.98 ± 0.21	6.54 ± 0.19	0.387	6.98 ± 0.14	6.42 ± 0.14	1.4*10^**−5**^	0.578
M-value (mg/min/kg)	4.93 ± 0.39	5.73 ± 0.43	**0.006**	4.98 ± 0.58	5.44 ± 0.41	0.229	0.764
Cholesterol (mmol/l)	5.16 ± 0.27	4.35 ± 0.26	**0.021**	5.24 ± 0.16	4.64 ± 0.18	**0.039**	0.883
LDL cholesterol (mmol/l)	3.25 ± 0.22	2.72 ± 0.22	**0.019**	3.40 ± 0.17	3.01 ± 0.17	**0.001**	0.638
HDL cholesterol (mmol/l)	1.13 ± 0.07	0.95 ± 0.05	**0.003**	1.09 ± 0.05	0.93 ± 0.03	**0.002**	0.111
Triglycerides (mmol/l)	1.72 ± 0.13	1.48 ± 0.14	0.182	1.64 ± 0.14	1.55 ± 0.15	1.000	0.106
Non-esterified free fatty acids (mmol/l)	0.67 ± 0.04	0.64 ± 0.05	1.000	0.73 ± 0.04	0.61 ± 0.04	**0.016**	0.425
C-reactive protein (mg/L)	2.17 ± 0.56	1.23 ± 0.33	**0.031**	2.09 ± 0.50	1.79 ± 0.40	1.000	0.836
IL-6 (pg/mL)	1.15 ± 0.25	0.89 ± 0.18	0.166	1.42 ± 0.32	1.40 ± 0.34	0.816	0.322
TNFα (pg/mL)	4.31 ± 0.68	4.37 ± 0.51	0.925	4.52 ± 0.60	3.76 ± 0.56	**0.016**	0.213
Urine nitrate/nitrite (μmol/l)	292.7 ± 65.8	423.8 ± 81.2	0.168	308.3 ± 66.0	246.8 ± 32.0	0.222	0.070

Values are means ± SEM. M-value obtained from hyperinsulinaemic–euglycaemic clamp was used for the assessment of the whole-body insulin sensitivity. Data were analysed with ANOVA repeated measures for each intervention group as well as for comparison between the groups (AP versus PP). Percentage weight change was used as a covariate in the model if significant interaction was found between the weight change and change in the parameter.

### 3.2 Oxidative and nitrosative stress markers

**Fig. 1:**
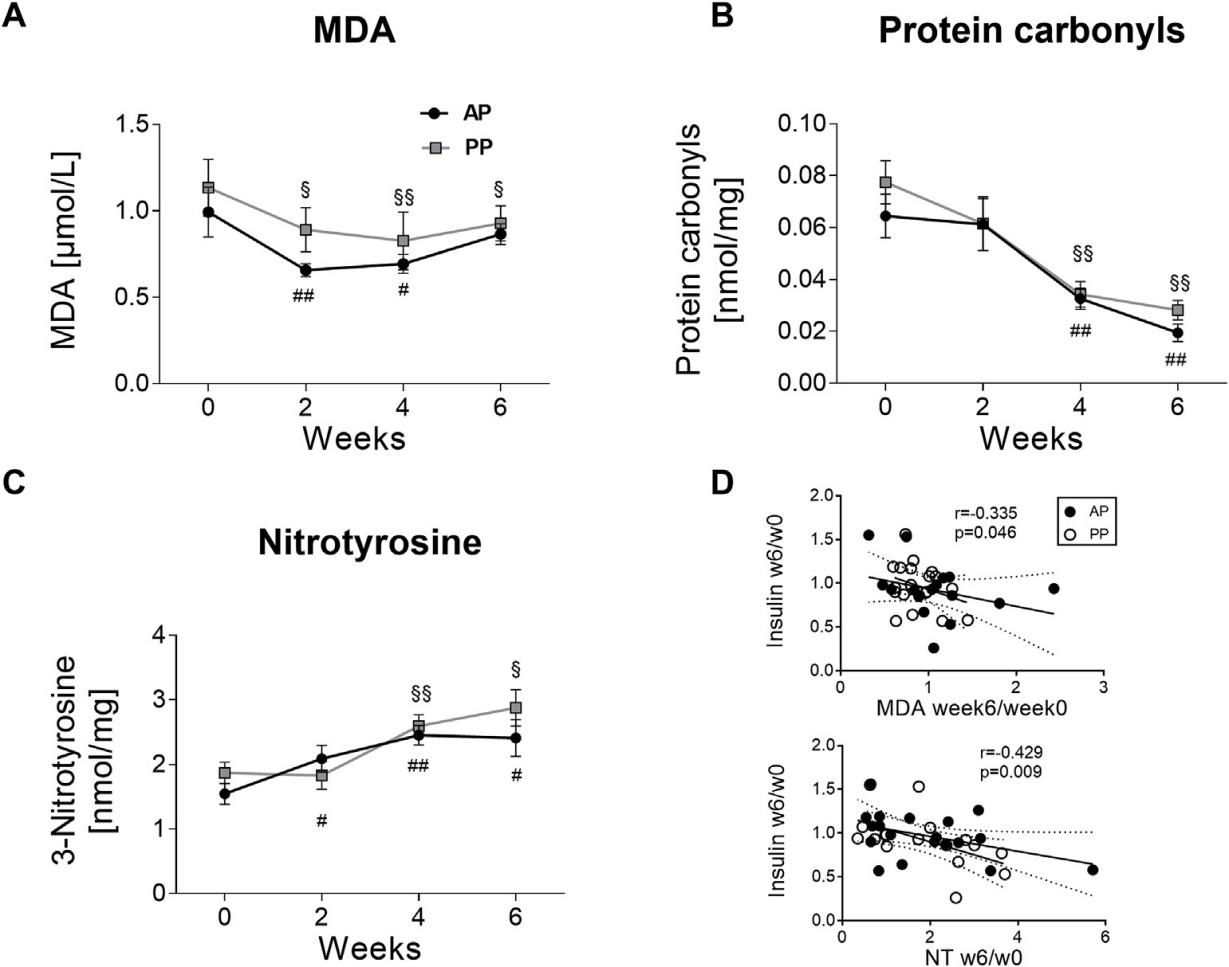
**Effects of animal and plant protein diets on oxidative and nitrosative stress markers.** Levels of (A) malondialdehyde (MDA); (B) protein carbonyls; (C) nitrotyrosine over 6 weeks of intervention. Values are means ± SEM. *p < 0.05, **p < 0.01 for AP vs. PP group; ^#^p < 0.05, ^# #^p < 0.01 vs. week 0 in the AP group; ^§^p < 0.05, ^§§^p < 0.01 vs. week 0 in the PP group. (D) Correlation between the relative change in MDA and nitrotyrosine (NT) from week 0 to week 6 with the relative change in fasting insulin. Black circles: AP group, white circles: PP group.

To investigate effects of high-protein diet on the oxidative stress, plasma levels of MDA and protein carbonyls were measured. In comparison with baseline, MDA levels was decreased after 2 and 4 weeks in the AP group and after 2, 4, and 6 weeks in the PP group without significant difference between groups (P_AP_ = 0.003, P_PP_ = 1.6 × 10^−4^, P_APvsPP_ = 0.469) (Fig. 1A). Protein carbonyls were strongly reduced after 4 and 6 weeks of both AP and PP diet (P_AP_ = 1.2 × 10^−4^, P_PP_ = 3.0 × 10^−5^, P_APvsPP_ = 0.745) (Fig. 1B). However, levels of nitrotyrosine (NT) often used as a nitrosative stress marker in observational studies [23] increased upon both AP and PP diets (P_AP_ = 0.005, P_PP_ = 0.004, P_APvsPP_ = 0.217) (Fig. 1C). Interestingly, dietary-induced changes of plasma MDA and NT from week 0 to week 6 correlated with the changes of fasting insulin (MDA: r = −0.335; P = 0.046; NT: r = −0.429; P = 0.009) (Fig. 1D) and HOMA-IR (MDA: r = −0.329; P = 0.050; NT: r = −0.436; P = 0.008), but not with changes of weight, fasting glucose, whole-body insulin sensitivity (M-value in clamps), NEFA, adiponectin, IL-6, TNFα, and iron. Further, changes of NT and HbA1c correlated inversely (r = −0.336; P = 0.041). Dietary-induced changes of protein carbonyls showed no associations with the above mentioned markers.

### 3.3 Dietary arginine intake and plasma levels

A recent study showed an association of high intake of the amino acid arginine with oxidative stress [9]. We hypothesized that a high arginine intake, which is also a precursor of nitric oxide (NO) might increase the production of peroxynitrite and NT synthesis. Indeed, in the present study, arginine intake was approximately 2-fold higher in both groups compared with baseline levels and not significantly different between diets (Table S2). However, arginine intake did not correlate with NT levels at week 6. Fasting plasma levels of arginine showed no difference between weeks 0 ad 6 and between diets (Table S3).

### 3.4 Expression of genes related to NO metabolism and oxidative stress pathways

We additionally analysed whether expression of enzymes involved in arginine/NO metabolism – arginase (Arg1), inducible NOS (iNOS), and endothelial NOS (eNOS) was affected by high-protein diets. However, mRNA expression levels in SAT showed no changes of these genes upon both diets (Fig. S2).

To gain further insight into the molecular mechanisms underlying metabolic changes upon high-protein diet, RNA-seq analysis of SAT samples was performed at baseline and after 6 weeks of intervention (n = 12 in the AP group and n = 15 in the PP group). In the AP group, 37 genes showed dietary induced expression changes, with 19 transcripts being upregulated and 18 being downregulated at week 6 in comparison with baseline (Table S4). Functional analysis revealed that these genes are involved in response to hyperoxia, cellular response to external stimulus, and protein metabolic processes. Further, translation, fatty acid biosynthesis, SREBP, and PPAR signaling were affected (Table 3), which is in agreement with previously published data [6]. We validated expression of two genes belonging to hyperoxia response pathway using qPCR: Fas cell surface death receptor (FAS) and elastin (ELN). PCR analysis confirmed a tendency to the upregulation of FAS and downregulation of ELN upon AP diet, but the changes did not reach statistical significance (Fig. S2). In the PP group, no transcripts showed dietary induced expression changes.

**Table 3:** Functional annotation of genes affected by AP diet.

Go term		p-value	q-value
**Biological processes**			
GO:0055093	response to hyperoxia	0.0012	0.1004
GO:0071496	cellular response to external stimulus	0.0016	0.1004
GO:0044763	single-organism cellular process	0.0052	0.2077
GO:0031668	cellular response to extracellular stimulus	0.0057	0.2017
GO:0044424	intracellular part	0.0062	0.1140
GO:0045137	development of primary sexual characteristics	0.0089	0.2017
GO:0042493	response to drug	0.0091	0.2017
GO:0019538	protein metabolic process	0.0097	0.2017
**Pathways**			
R-HSA-418360	Platelet calcium homeostasis	0.0017	0.0540
De novo fatty acid biosynthesis	De novo fatty acid biosynthesis	0.0036	0.0540
R-HSA-75105	Fatty Acyl-CoA Biosynthesis	0.0052	0.0540
R-HSA-72695	Formation of the ternary complex, and subsequently, the 43 S complex	0.0060	0.0540
R-HSA-72649	Translation initiation complex formation	0.0075	0.0540
R-HSA-72702	Ribosomal scanning and start codon recognition	0.0075	0.0540
R-HSA-72662	Activation of the mRNA upon binding of the cap-binding complex and eIFs, and subsequent binding to 43 S	0.0077	0.0540
WP3942	PPAR signaling pathway	0.0087	0.0540
WP1982	Sterol Regulatory Element-Binding Proteins (SREBP) signaling	0.0087	0.0540
path:hsa 03320	PPAR signaling pathway - *Homo sapiens* (human)	0.0097	0.0543

Transcripts showed significant changes after a false-discovery rate correction (p < 0.05) were subjected for gene ontology and pathway analyses. Functional annotation was done with ConsensusPathDB.

### 3.5 Nitrate/nitrite levels in urine

We further investigated the levels of NO metabolites excreted in urine by the measurement of nitrate/nitrite concentrations in 24 h urine samples as described [24]. No significant changes of urine nitrate/nitrite levels after 6 weeks of AP or PP intervention compared to baseline and no difference between groups were found (Table 2) with high interindividual variability of AP effects (Fig. S3). Similar results were obtained after the normalization to urinary creatinine levels (data not shown).

### 3.6 Antioxidant vitamins and carotenoids

**Fig. 2:**
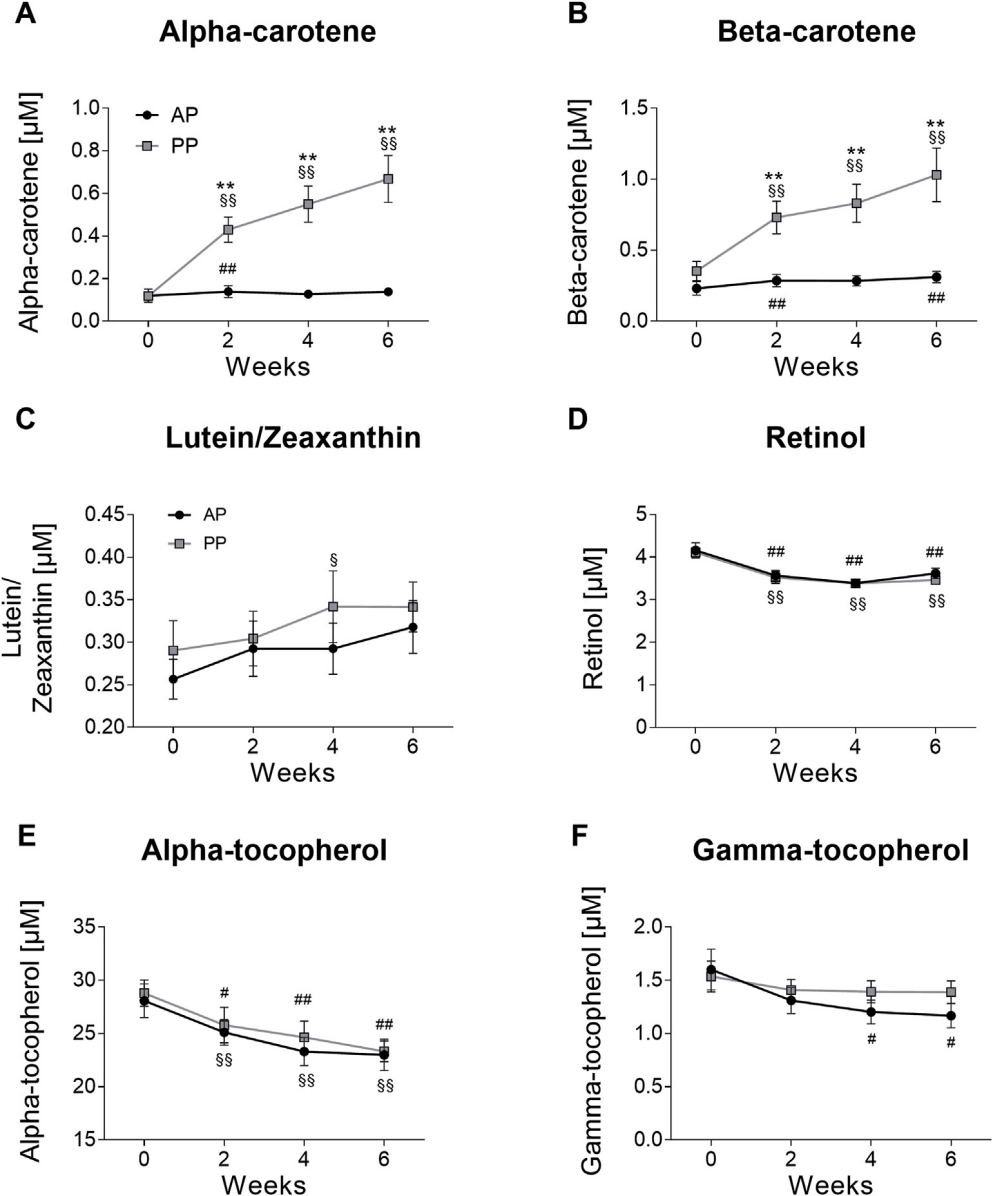
**Effects of animal and plant protein diets on plasma antioxidant vitamins and carotenoids.** Levels of (A) alpha-carotene; (B) beta-carotene; (C) lutein/zeaxanthin; (D) retinol; (E) alpha-tocopherol, and (F) gamma-tocopherol over 6 weeks of intervention. Values are means ± SEM. *p < 0.05, **p < 0.01 for AP vs. PP group; #p < 0.05, # #p < 0.01 vs. week 0 in the AP group; §p < 0.05, §§p < 0.01 vs. week 0 in the PP group.

We finally analysed plasma levels of antioxidant vitamins and carotenoids, which might contribute to observed changes of oxidative stress markers. Levels of alpha-carotene dramatically increased upon PP diet, whereas AP group showed minor increase over 6 weeks (P_AP_ = 0.013, P_PP_ = 7.1 × 10^−12^, P_APvsPP_ = 4.0 × 10^−8^) (Fig. 2A). Beta-carotene levels showed similar changes (P_AP_ = 0.046, P_PP_ = 5.8 × 10^−8^, P_APvsPP_ = 0.013) (Fig. 2B). PP, but not AP, also slightly increased lutein/zeaxanthin levels without difference between diets (P_AP_ = 0.109, P_PP_ = 0.044, P_APvsPP_ = 0.820) (Fig. 2C). Beta-cryproxanthin and lycopene showed no dietary-induced changes (data not shown). However, both AP and PP diets resulted in reduction of plasma retinol (P_AP_ = 4.5 × 10^−5^, P_PP_ = 7.1 × 10^−7^, P_APvsPP_ = 0.726) (Fig. 2D) and alpha-tocopherol levels (P_AP_ = 1.1 × 10^−5^, P_PP_ = 4.6 × 10^−7^, P_APvsPP_ = 0.834) (Fig. 2E). Gamma-tocopherol levels also decreased upon AP diet, but not in the PP group (P_AP_ = 0.004, P_PP_ = 0.602, P_APvsPP_ = 0.407) (Fig. 2F). No decrease of plasma tocopherols was found after the normalization with regard to triglyceride levels (Fig. S4).

We hypothesized that differences in plasma antioxidant levels might be explained by the dietary micronutrient intake. Indeed, PP diet provided 1.5-fold more beta-carotene than AP diet (p = 5.0 × 10^−4^), although consumption of beta-carotene upon both diets was higher than at baseline (Table 1). Dietary intake of retinol decreased, whereas alpha-tocopherol and vitamin C intake similarly increased upon both diets (Table 1).

## 4 Discussion

In this study, we compared effects of diets high in animal or plant protein on markers of oxidative stress and antioxidant status in subjects with T2DM for the first time. We found that both AP and PP diets similarly reduced levels of MDA and protein carbonyls over 6 weeks of intervention, accompanied by a marked improvements of glycemic control, decrease of blood lipids, blood pressure, and inflammatory cytokines [3,6]. However, plasma NT increases upon both diets. Notably, dietary-induced changes of plasma MDA and NT were associated with changes of fasting insulin, HOMA-IR, and HbA1c, suggesting an important role of oxidative damage in the regulation of beta-cell function and insulin resistance via multiple mechanisms [10,11].

Our data on MDA and protein carbonyls are in agreement with a study described by Kitabchi et al. who showed a stronger reduction of ROS levels and MDA and larger improvement of insulin sensitivity, cardiovascular risk factors upon hypocaloric diet rich in animal protein in comparison with a high-carbohydrate diet [8]. Nevertheless, literature data concerning effects of animal and plant protein are very contradictory. In particular, high meat consumption, especially red and processed meat, typical for Western-type diets, lead to an elevated production of ROS [25,26], which is associated with an increased risk of T2DM, insulin resistance, and other cardiometabolic dysfunctions as shown in epidemiological studies [27]. In contrast, replacement of red meat with soy protein reduced plasma MDA and increased plasma total antioxidant capacity [28], although beneficial effects of plant proteins were not confirmed by another group [29].

Amino acid composition of dietary protein is suggested to strongly affect its metabolic effects [30]. PP usually contains lower levels of the branched chain amino acids and methionine as compared with AP [31], being also a result in the present study. In accordance with epidemiological studies on red meat intake, a high-methionine diet in rats showed an increased level of MDA and NT in the liver [32]. In our study, AP diet was rich in white meat and dairy foods (and might be therefore designated as casein-rich diet [30]), whereas PP diet consisted mainly of pea protein-based food items. Unexpectedly, and despite of difference in the amino acid composition, both diets not only similarly reduced levels of MDA and protein carbonyls, but also similarly increased plasma NT levels.

To explain the NT changes, we focussed on the metabolism of arginine, which is a main substrate of NO synthesis and its intake was similarly 2-fold increased upon both AP and PP diet compared with baseline. Whereas some studies showed beneficial effects of increased arginine intake on endothelial function and insulin sensitivity via NO production [33,34], several research groups found adverse outcomes upon chronic arginine supplementation [35]. A recent study of Carvalho et al. revealed an association of arginine intake with oxidative stress marker MDA [9]. We hypothesized that high arginine intake might increase a NO production and synthesis of peroxynitrite and corresponding tyrosine nitration. Although fasting plasma levels of arginine did not differ between weeks 0 and 6, this can be explained by rapid postprandial intake of arginine into the cells [6], following by its decrease in circulation to the fasting levels.

Intracellularly, arginine can be used for protein synthesis or metabolized by NOS to generate nitric oxide (NO*) and l-citrulline, or cleaved by arginases to ornithine and urea [36]. We therefore investigated whether increased arginine intake upregulate enzymes involved in arginine/NO metabolism – Arg1, iNOS and eNOS – and in this way lead to the excessive NO production. PCR analysis of these enzymes did not reveal expression changes upon AP and PP diet at the mRNA level. However, we cannot exclude that regulation of translation or activity of these enzymes occur upon the high arginine intake [37].

Interestingly, RNA-Seq analysis confirmed the alterations of hyperoxia response pathway upon AP diet which is an absolute novel finding concerning the effect of the high-protein diet. For two genes involved in this pathway – FAS (death receptor associated with hyperoxia-induced apoptosis) and ELN (connective tissue protein regulated by hyperoxia) – PCR analysis confirmed a tendency revealed by RNA-Seq analysis. Activation of the FAS-mediated apoptosis might be induced by the increased NT formation ^39^ especially upon hyperglycemia characteristic for T2DM subjects ^40^. However, we cannot exclude that the downregulation of ELN in SAT in our study resulted from the minor weight loss upon high-protein diets [38].

In despite of our hypothesis about excessive NO production and literature data [24], we did not find significant alterations of NO metabolites (nitrates and nitrites) in urine upon AP or PP diets rich in arginine. Urine nitrate/nitrite levels can also mirror the dietary intake of nitrates and nitrites [39], which might cause the increased NT production according to some data [40]. However, lack of the increase of urine nitrate/nitrite levels suggests that NT increase also cannot be explained by dietary intake of nitrates and nitrites. Interestingly, individual changes of urine NO metabolites were heterogenous, especially in the AP group, which might result from interindividual differences in the nitrate/nitrite intake or in the response to the arginine-rich diet. Taken together, our data cannot explain the mechanism of the NT increase upon high-protein diets which therefore needs investigation in the future studies.

Notably, effect of different dietary proteins may relate not only to differences in amino acid composition of the proteins *per se*, but also to other dietary components, such as increased intake of fibre or phytochemical antioxidants in plant food products [31]. We therefore tested the hypothesis that plasma levels of antioxidant vitamins and carotenoids might contribute to changes of oxidative stress in our study. Indeed, we found strong increases of plasma alpha- and beta-carotene levels and lutein/zeaxanthin levels upon PP diet, which correspond to the increased carotenoid intake upon PP diet. Similarly, decreased retinol plasma levels are in agreement with its lower dietary intake compared to the baseline which was only 40–50% of the reference value (0.8–1.0 mg/day retinol equivalent) [41]. High intake of carotenoids (a precursor of vitamin A) upon PP diet was still not sufficient to compensate a reduction of plasma retinol levels. Interestingly, alpha- and gamma-tocopherol in plasma slightly decreased upon both diets despite of the increased dietary intake compared to the baseline which was 2-fold above German reference values (11–14 mg/day) [41]. This might be explained by the decrease of fat intake and blood lipids, which might aggravate the absorption of fat-soluble vitamins. However, this phenomenon might be also explained by some (hidden) redox processes consuming tocopherols. Vitamin C intake was also strongly increased upon both diets compared to the baseline, which might be explained by elevated fruit and vegetable consumption during dietary intervention. Thus, dietary induced changes of plasma antioxidant vitamins cannot explain similar improvement of oxidative stress markers in AP and PP groups. Mechanisms of this phenomenon also need future investigation. In particular, activity of antioxidant enzymes such as superoxide dismutase, catalase, and glutathione peroxidase might be differently affected by different dietary proteins [42].

## 5 Conclusions

In conclusion, both AP and PP diets similarly reduce oxidative stress markers MDA and protein carbonyls, but lead to an increase of the nitrosative stress marker NT in diabetic subjects, which were related to changes of fasting insulin and insulin resistance. Our findings suggest the effective use of high-protein diets for diabetes prevention and treatment. Molecular mechanisms of oxidative stress regulation by different dietary proteins have to be further elucidated in future studies, focusing especially on the responsibilities of endogenous mechanisms and exogenous dietary uptake of potential antioxidants.

## Author contributions

T.G. and A.F·H.P. were responsible for the conception and design of the study. S.S., S·H., M.M., N.R., and D.W. conducted the study. O.P-R., M.M., D.W., S·S and D.S.-F. contributed to acquisition of data, review of data, analysis of data, and discussion of data. O.P-R., A.F·H·P., and T.G. were responsible for drafting of the manuscript. O.P-R., M.M., N.R., J.R., S.R., A.F·H·P., and T.G. contributed to the critical revision of the manuscript for important intellectual content. O.P-R. is the guarantor of this work and, as such, had full access to all the data in the study and takes responsibility for the integrity of the data and the accuracy of the data analysis.

## Funding

The study was supported by a grant from the German Federal Ministry of Food and Agriculture (BLE Grant No. 2815407110 to AFHP) and by a grant of the German Center for Diabetes Research (DZD) “FGF21 as a novel bidirectional regulator of protein preference and aversion in humans and mice: demonstration and regulatory mechanisms and clinical implications”. The funding source had no role in study design, data collection, analysis or interpretation, report writing, or the decision to submit this paper for publication.

## Declaration of competing interest

The authors have no conflict of interest to declare.
