# Supplementary material for: Effects of diets high in animal or plant protein on oxidative stress in individuals with type 2 diabetes: A randomized clinical trial
Source: Redox Biol. 2019 Nov 28;29:101397. doi: 10.1016/j.redox.2019.101397 (PMC6909130; doi:10.1016/j.redox.2019.101397)
Supplement: Multimedia component 1 [file mmc1.docx]

**Table S1. Primers used for real-time PCR.**

| **Gene name** | **Gene symbol** | **Gene ID** | **Primer sequence** | |
| --- | --- | --- | --- | --- |
|  |  |  | **forward** | **reverse** |
| Inducible NO synthase | *iNOS* | 4843 | GGTGGAAGCGGTAACAAAGG | TGCTTGGTGGCGAAGATGA |
| Endothelial NO synthase | *eNOS* | 4846 | TGGACCTGGATACCCGGAC | TGGTGACTTTGGCTAGCTGGT |
| Arginase 1 | *ARG1* | 383 | AGCGCCAAGTCCAGAACCATA | TCAAGCAGACCAGCCTTTCTCA |
| Fas cell surface death receptor | *FAS* | 355 | TGAAGGACATGGCTTAGAAGTG | GGTGCAAGGGTCACAGTGTT |
| Elastin | *ELN* | 2006 | GGCCATTCCTGGTGGAGTTCC | AACTGGCTTAAGAGGTTTGCCTCCA |
| Glucuronidase, beta | *GUSB* | 2990 | CTCATTTGGAATTTTGCCGATT | CCGAGTGAAGATCCCCTTTTTA |

**Table S2. Dietary intake of amino acids and iron.**

| **Variables** | **AP** | | **PP** | |
| --- | --- | --- | --- | --- |
|  | **Week 0** | **Week 6** | **Week 0** | **Week 6** |
| Alanine (mg) | 4632.0 ± 325.3 | 7918.6 ± 300.5** | 4074.1 ± 274.6 | 6683.1 ± 340.2**^#^ |
| Arginine (mg) | 5266.4 ± 356.2 | 8933.7 ± 331.0** | 4571.9 ± 271.7 | 9980.0 ± 490.1** |
| Asparagin (mg) | 8524.6 ± 599.1 | 15507.5 ± 532.3** | 7400.3 ± 469.6 | 14734.4 ± 719.5** |
| Cysteine (mg) | 1304.0 ± 69.7 | 1821.1 ± 71.8** | 1119.1 ± 62.1^#^ | 2042.9 ± 102.7** |
| Glutamine (mg) | 19400.0 ± 1162.0 | 36870.9 ± 1199.6** | 16498.8 ± 815.0 | 33911.8 ± 1781.3** |
| Histidine (mg) | 2733.1 ± 209.0 | 5493.2 ± 197.1** | 2318.4 ± 145.2 | 4019.9 ± 220.8**^##^ |
| Isoleucine (mg) | 4618.4 ± 329.6 | 9415.5 ± 296.2** | 3869.2 ± 234.6 | 7302.0 ± 373.7**^##^ |
| Leucine (mg) | 7555.3 ± 526.1 | 15868.2 ± 491.1** | 6313.2 ± 364.5 | 14374.7 ± 710.0** |
| Lysine (mg) | 6520.8 ± 521.1 | 13887.9 ± 458.0** | 5456.3 ± 364.0 | 9617.5 ± 512.5**^##^ |
| Methionine (mg) | 2127.5 ± 160.6 | 4483.5 ± 137.1** | 1769.3 ± 113.2 | 2995.9 ± 156.7**^##^ |
| Phenylalanine (mg) | 4280.5 ± 282.8 | 8446.1 ± 263.7** | 3633.3 ± 204.2 | 7853.5 ± 404.4** |
| Proline (mg) | 6934.6 ± 469.3 | 14930.2 ± 474.3** | 6023.5 ± 305.3 | 11135.0 ± 612.0**^##^ |
| Serine (mg) | 4706.7 ± 312.1 | 9356.8 ± 287.3** | 4025.6 ± 228.6 | 8080.9 ± 407.9**^##^ |
| Threonine (mg) | 3954.7 ± 283.3 | 7827.4 ± 250.4** | 3348.1 ± 204.0 | 5978.6 ± 310.7**^##^ |
| Tryptophan (mg) | 1125.9 ± 75.6 | 2205.6 ± 65.9** | 986.9 ± 58.1 | 1803.9 ± 87.3**^##^ |
| Tyrosine (mg) | 3439.2 ± 256.6 | 7631.7 ± 235.2** | 2891.7 ± 169.9 | 5849.4 ± 305.7**^##^ |
| Valine (mg) | 5314.1 ± 381.6 | 10886.1 ± 336.8** | 4565.1 ± 266.7 | 8218.9 ± 424.1**^##^ |
| Fe (mg) | 14.2 ± 4.1 | 17.0 ± 1.0* | 13.5 ± 4.2 | 14.3 ± 0.8 |

Values are means ± SEM. n_AP_=18, n_PP_=19. * p<0.05, ** p<0.01 week 6 vs. week 0; # p<0.05, ## p<0.01 AP vs. PP at week 0 or at week 6.

**Table S3. Fasting plasma levels of amino acids and serum iron.**

| **Variables** | **AP** | | **PP** | |
| --- | --- | --- | --- | --- |
|  | **Week 0** | **Week 6** | **Week 0** | **Week 6** |
| Alanine (μM) | 198.6 ± 15.9 | 175.8 ± 11.7 | 190.8 ± 14.0 | 172.8 ± 9.4 |
| Arginine (μM) | 49.9 ± 2.8 | 47.1 ± 1.9 | 50.8 ± 4.2 | 48.0 ± 3.5 |
| Asparagine (μM) | 23.3 ± 1.9 | 22.7 ± 1.4 | 22.2 ± 1.9 | 23.4 ± 2.1 |
| Glutamine (μM) | 960.3 ± 64.1 | 853.9 ± 31.9 | 912.1 ± 52.6 | 898.7 ± 51.5 |
| Histidine (μM) | 43.4 ± 2.1 | 45.5 ± 2.5 | 44.1 ± 2.5 | 41.9 ± 2.9 |
| Isoleucine (μM) | 83.9 ± 5.3 | 80.6 ± 2.7 | 75.8 ± 4.8 | 76.7 ± 5.0 |
| Leucine (μM) | 189.2 ± 11.6 | 173.1 ± 4.4 | 174.7 ± 10.1 | 170.1 ± 9.0 |
| Lysine (μM) | 884.9 ± 64.9 | 931.3 ± 46.6 | 848.4 ± 34.5 | 808.0 ± 42.9 |
| Methionine (μM) | 25.4 ± 1.1 | 23.3 ± 0.9 | 22.4 ± 1.3 | 22.7 ± 1.2 |
| Phenylalanine (μM) | 44.4 ± 2.0 | 43.7 ± 1.4 | 43.9 ± 2.3 | 43.2 ± 2.4 |
| Serine (μM) | 48.8 ± 3.4 | 55.8 ± 2.8 | 53.6 ± 6.3 | 49.1 ± 4.9 |
| Taurine (μM) | 43.3 ± 4.6 | 33.2 ± 2.1 * | 39.9 ± 2.9 | 31.6 ± 2.0 * |
| Tryptophan (μM) | 8.0 ± 0.4 | 8.1 ± 0.6 | 7.6 ± 0.5 | 7.1 ± 0.4 |
| Tyrosine (μM) | 46.7 ± 2.1 | 44.7 ± 2.4 | 44.4 ± 2.4 | 43.1 ± 2.1 |
| Valine (μM) | 94.7 ± 6.6 | 101.8 ± 4.2 | 94.2 ± 5.2 | 94.2 ± 4.7 |
| Fe (mg/L) | 1.34 ± 0.09 | 1.34 ± 0.21 | 1.80 ± 0.40 | 1.20 ± 0.05 |

Values are means ± SEM. n_AP_=18, n_PP_=19. * p<0.05, ** p<0.01 week 6 vs. week 0.

**Table S4. List of genes up- or downregulated by AP diet**

| **Gene symbol** | **Gene name** | **Fold change** | **P-value** | **Padj** | **Regulation** |
| --- | --- | --- | --- | --- | --- |
| KIAA1324 | KIAA1324 | 2.74 | 2.06E-05 | 3.79E-02 | up |
| ATP2A3 | ATPase sarcoplasmic/endoplasmic reticulum Ca2+ transporting 3 | 2.19 | 6.39E-06 | 1.76E-02 | up |
| P2RX1 | purinergic receptor P2X 1 | 2.02 | 1.37E-07 | 1.26E-03 | up |
| RABGAP1L | RAB GTPase activating protein 1 like | 1.99 | 1.08E-05 | 2.71E-02 | up |
| LPCAT1 | lysophosphatidylcholine acyltransferase 1 | 1.98 | 5.81E-06 | 1.68E-02 | up |
| CPVL | carboxypeptidase, vitellogenic like | 1.68 | 2.58E-05 | 4.17E-02 | up |
| ADGRE5 | adhesion G protein-coupled receptor E5 | 1.68 | 1.88E-06 | 7.96E-03 | up |
| TBPL1 | TATA-box binding protein like 1 | 1.62 | 2.75E-06 | 9.47E-03 | up |
| RPS13 | ribosomal protein S13 | 1.55 | 1.55E-06 | 7.11E-03 | up |
| CTDSP1 | CTD small phosphatase 1 | 1.54 | 2.23E-05 | 3.83E-02 | up |
| FAS | Fas cell surface death receptor | 1.48 | 2.36E-05 | 3.93E-02 | up |
| IL18R1 | interleukin 18 receptor 1 | 1.45 | 2.50E-08 | 5.32E-04 | up |
| TMEM259 | transmembrane protein 259 | 1.43 | 2.78E-05 | 4.37E-02 | up |
| XPO6 | exportin 6 | 1.38 | 2.51E-06 | 9.47E-03 | up |
| NBPF26 | NBPF member 26 | 1.37 | 8.31E-08 | 1.14E-03 | up |
| CHD3 | chromodomain helicase DNA binding protein 3 | 1.36 | 8.15E-07 | 4.99E-03 | up |
| KDM6B | lysine demethylase 6B | 1.34 | 1.37E-07 | 1.26E-03 | up |
| KMT2B | lysine methyltransferase 2B | 1.17 | 5.67E-06 | 1.68E-02 | up |
| DYRK1A | dual specificity tyrosine phosphorylation regulated kinase 1A | 1.15 | 1.42E-05 | 3.26E-02 | up |
| EIF2S2 | eukaryotic translation initiation factor 2 subunit beta | 0.86 | 1.21E-06 | 6.04E-03 | down |
| TMED1 | transmembrane p24 trafficking protein 1 | 0.83 | 3.02E-05 | 4.49E-02 | down |
| LINC00667 | long intergenic non-protein coding RNA 667 | 0.79 | 2.21E-05 | 3.83E-02 | down |
| DPH3 | diphthamide biosynthesis 3 | 0.78 | 9.36E-06 | 2.45E-02 | down |
| PDSS2 | decaprenyl diphosphate synthase subunit 2 | 0.77 | 5.74E-06 | 1.68E-02 | down |
| KLHDC8B | kelch domain containing 8B | 0.73 | 1.70E-05 | 3.60E-02 | down |
| DBI | diazepam binding inhibitor, acyl-CoA binding protein | 0.70 | 6.81E-09 | 3.75E-04 | down |
| NRBP2 | nuclear receptor binding protein 2 | 0.69 | 1.92E-05 | 3.65E-02 | down |
| ELN | elastin | 0.69 | 1.13E-05 | 2.71E-02 | down |
| LOX | lysyl oxidase | 0.68 | 1.77E-05 | 3.61E-02 | down |
| CDKN2B | cyclin dependent kinase inhibitor 2B | 0.64 | 3.46E-07 | 2.38E-03 | down |
| SNF8 | SNF8, ESCRT-II complex subunit | 0.63 | 1.04E-06 | 5.73E-03 | down |
| NNMT | nicotinamide N-methyltransferase | 0.62 | 2.93E-05 | 4.48E-02 | down |
| NEXN | nexilin F-actin binding protein | 0.58 | 2.90E-08 | 5.32E-04 | down |
| ME3 | malic enzyme 3 | 0.58 | 1.87E-05 | 3.65E-02 | down |
| ASAH1 | N-acylsphingosine amidohydrolase 1 | 0.58 | 1.49E-05 | 3.27E-02 | down |
| SYPL1 | synaptophysin like 1 | 0.48 | 2.35E-07 | 1.85E-03 | down |
| SCD | stearoyl-CoA desaturase | 0.45 | 2.69E-06 | 9.47E-03 | down |
