# Supplementary material for: Effects of diets high in animal or plant protein on oxidative stress in individuals with type 2 diabetes: A randomized clinical trial
Source: Redox Biol. 2019 Nov 28;29:101397. doi: 10.1016/j.redox.2019.101397 (PMC6909130; doi:10.1016/j.redox.2019.101397)
Supplement: Multimedia component 2 [file mmc2.pdf]

Figure S1

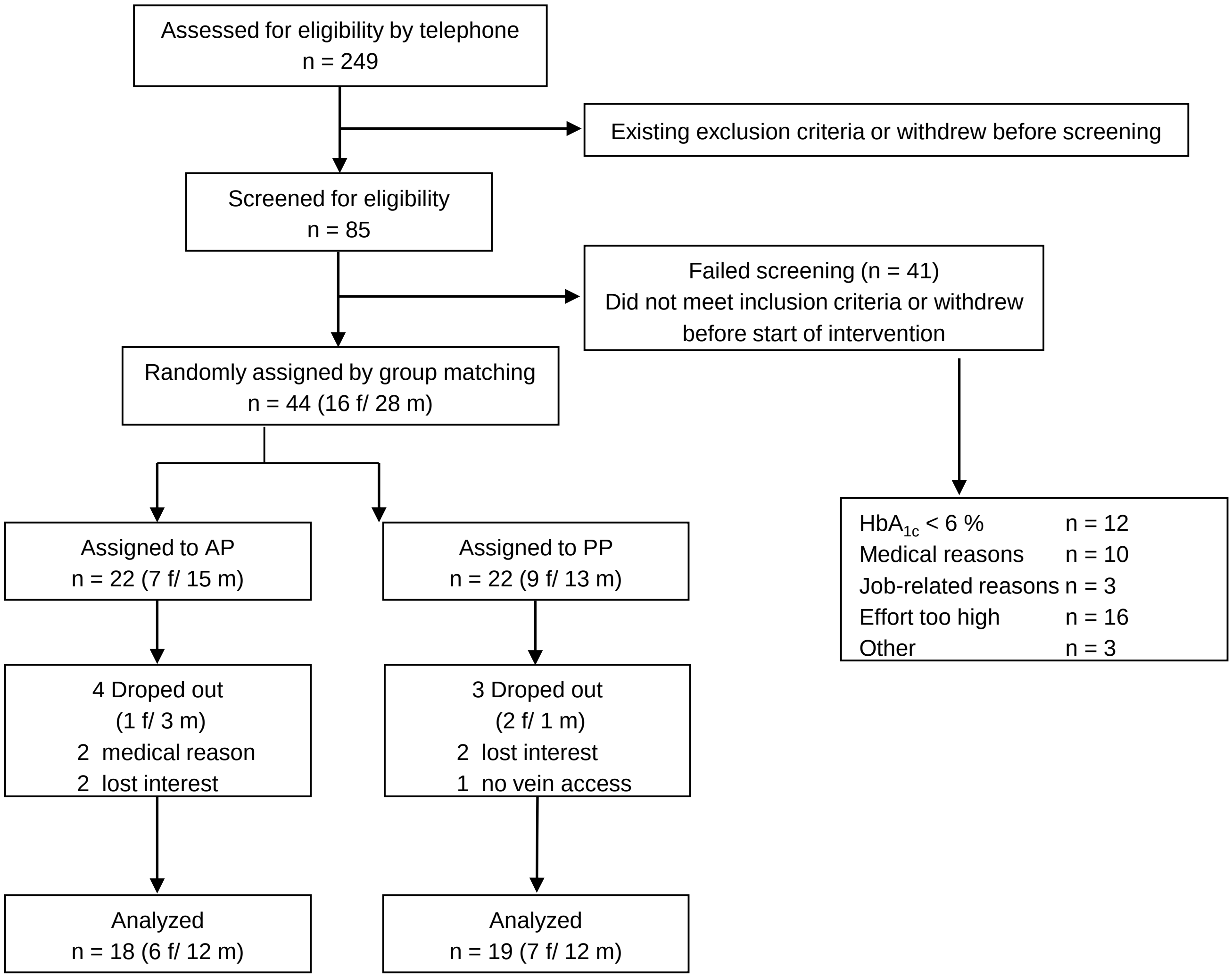

Figure S1. Flowchart of the subject recruitment for the trial.
AP – animal protein; PP – plant protein.

Figure S2

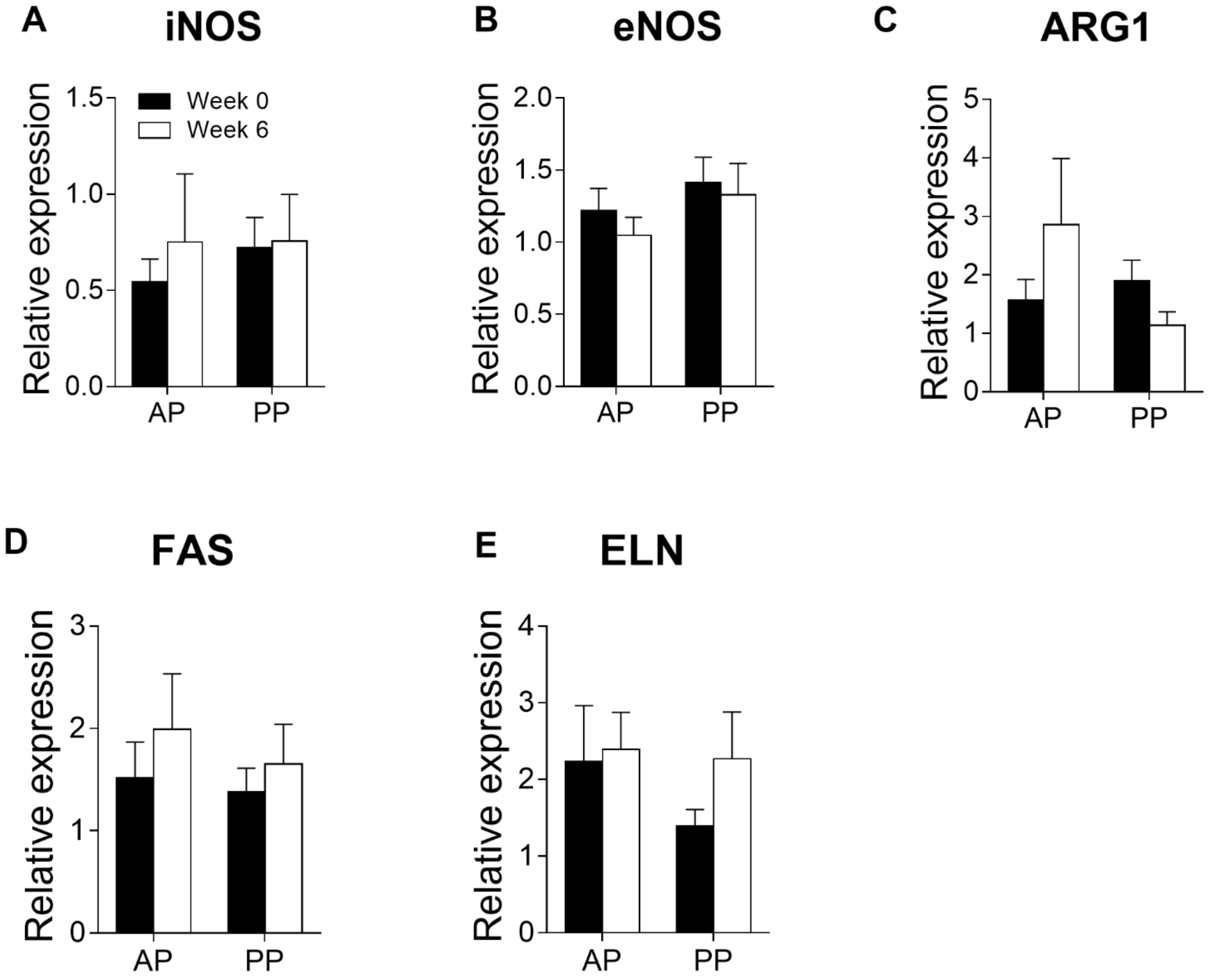

Figure S2. Effects of animal and plant protein diets on the gene expression in adipose tissue. Levels of mRNA for (A) inducible NO synthase (iNOS); (B) endothelial NO synthase (eNOS); (C) arginase 1 (Arg1); (D) Fas cell surface death receptor (FAS), and (E) elastin (ELN) in subcutaneous adipose tissue at baseline and after 6 weeks of dietary intervention. Values are means ± SEM. *$p<0.05$ for week 6 vs. week 0.

Figure S3

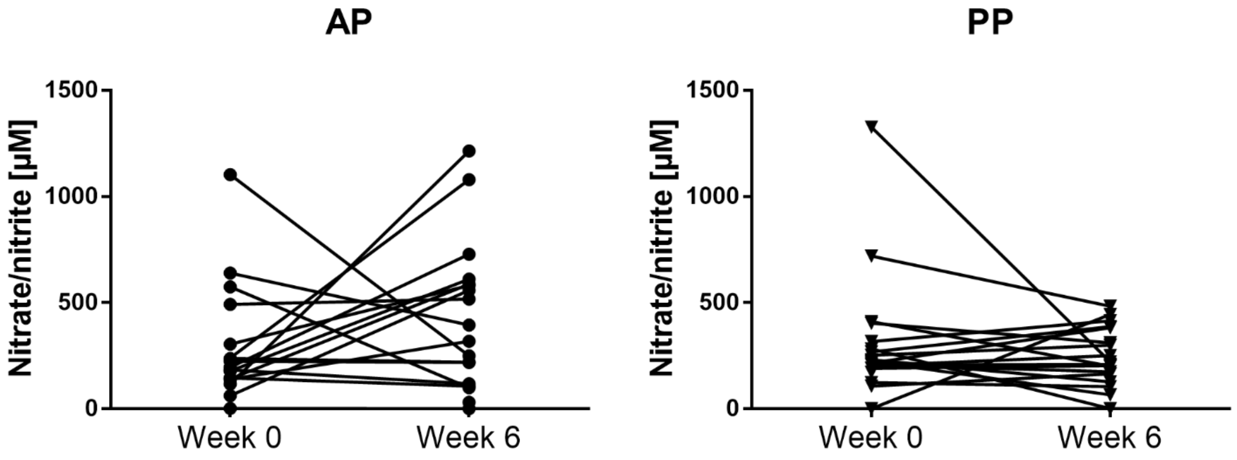

Figure S3. Individual changes in urine NO metabolite levels. AP – animal protein, PP – plant protein group. nAP = 18, nPP = 19.

Figure S4

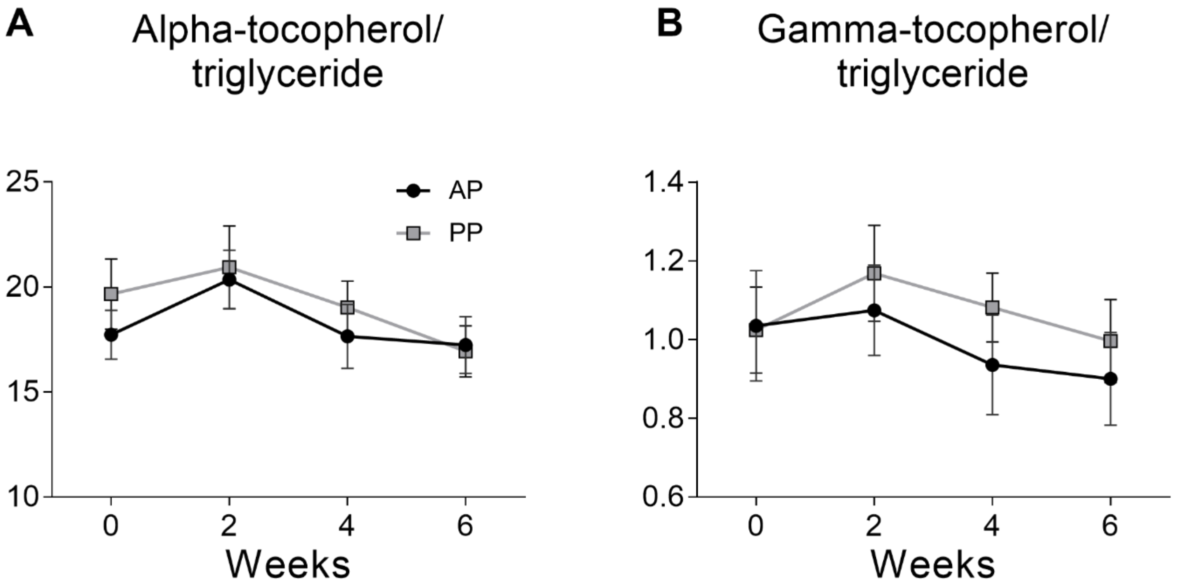

Figure S4. Plasma alpha-tocopherol (A) and gamma-tocopherol (B) normalised to the triglyceride levels over 6 weeks of intervention. Values are means ± SEM. *p<0.05, **p<0.01 for AP vs. PP group; $^{\#}$p<0.05, $^{\#\#}$p<0.01 vs. week 0 in the AP group; §p<0.05, §§p<0.01 vs. week 0 in the PP group.
